# The Impact of Emotion on Musical Long-Term Memory

**DOI:** 10.3389/fpsyg.2020.02110

**Published:** 2020-08-31

**Authors:** Clémence Nineuil, Delphine Dellacherie, Séverine Samson

**Affiliations:** ^1^Univ. Lille, ULR 4072 ‐ PSITEC ‐ Psychologie: Interactions, Temps, Emotions, Cognition, Lille, France; ^2^Department of Pediatric Neurology, University Hospital of Lille, Lille, France; ^3^AP-HP, GH Pitié-Salpêtrière-Charles Foix, Unité d’Epileptologie, Paris, France

**Keywords:** emotion, musical memory, consolidation, valence, arousal

## Abstract

The influence of emotional dimensions such as arousal and valence on memory has been a topic of particularly intense inquiry. As stimuli go, music is capable of provoking strong emotional responses from listeners, which can in turn influence memory. However, few studies have examined the effect of musical emotions on memory, and even fewer the effect of valence and arousal. In order to shed light on the ways in which emotional dimensions affect musical memory as study-test delay intervals increase, we tested recognition after a short delay and after a long delay. In line with the literature, we hypothesized an emotional enhancement of music memory induced by post-encoding processes leading to better recognition of musical excerpts in delayed condition, as compared to the immediate condition. The effects of arousal and valence were expected to become exaggerated after a long delay. We also predicted that the two emotional dimensions would be differently affected by the study-test intervals. Our results showed that the emotional enhancement of memory depends upon the valence, with remembering of positive and negative stimuli being differently affected by the duration of the study-test delay interval. Furthermore, our data demonstrated that musical excerpts were better recognized after a long delay than after a short delay, illustrating that memory consolidation for musical information is taking place during the long study-test interval. Moreover, musical memory consolidation is strongly related to the characteristics of the positive valence, which have been discussed in relation to its pleasantness. This original finding provides new insights into the modulatory effects of emotional valence on memory consolidation and could offer promising therapeutic possibilities for the rehabilitation of memory disorders.

## Introduction

Emotional events from our life are more likely to be later recollected than similar, non-emotional events. This emotional enhancement of memory has been extensively studied using words and pictures (for review, see Yonelinas and Ritchey, 2015). Fewer studies have however examined the influence of emotion on musical memory. And yet, the emotional power of music is well-established (Koelsch, 2014), and musical memory may in fact benefit from such emotional enhancements, explaining why certain pieces of music frequently become unforgettable. Is this related to the particularly efficient post-encoding processes involved in memorizing musical information? In order to investigate this question, we examined musical memory abilities in adult listeners by manipulating the emotional characteristics of musical excerpts and the lengths of time separating the study phase and a recognition test.

Emotion theorists often take the position that affective experience can be described according to two orthogonal dimensions, namely arousal and valence (Russell, 1980). Arousal refers to a continuum ranging from calm to excitement, whereas valence is measured along a continuum ranging from positive to negative. The impact of these emotional dimensions on different forms of memory, including declarative (explicit) memory, has been explored at great length. In particular, evidence has shown that high-arousal information is better remembered than low-arousal information (for review, see Hamann, 2001) in immediate (Bradley et al., 1992) or delayed memory tests administered after 1 day (Hu et al., 2006), a year (Bradley et al., 1992) or several years (Wagner et al., 2006). By increasing attention and elaboration at time of encoding, high-arousal stimuli are thought to be more deeply processed than low-arousal stimuli. Their memory trace would be subsequently enhanced by post-encoding processes, including stress hormone release, leading to better consolidation over a long period of time (Hamann, 2001; Sharot and Phelps, 2004; Payne et al., 2008; Sharot and Yonelinas, 2008). Emotional valence has also been reported to influence memory, with stimuli with a negative or positive valence being better memorized than neutral stimuli (Ely et al., 1999; Cabeza and Dolcos, 2002; Mickley and Kensinger, 2008). Furthermore, remembering negative information yielded even higher memory performance than remembering positive information, whether using pictures (Comblain et al., 2004; Waring and Kensinger, 2009) or words (Inaba et al., 2005; for review, see Kensinger, 2007). This effect would persist over days (Denburg et al., 2003; Kensinger, 2007; Waring and Kensinger, 2009) or weeks (Ely et al., 1999; Ochsner, 2000; Pierce and Kensinger, 2011), suggesting a negativity bias in emotional memory, at least in young adults. Few studies have conjointly manipulated arousal and valence within a single experiment. In some cases, valence and arousal had their own respective effects on memory. Arousing pictures were better remembered than non-arousing ones and negative pictures were better memorized than positive ones (Comblain et al., 2004). In other investigations, the effect of arousal on memory performance was found to be modulated by valence (Kensinger, 2008; Li et al., 2018). However, although studies show interaction effects, the nature of the interaction differs. This interaction and its impact on memory performance are still debated in the behavioral literature (Ochsner, 2000; Dolcos et al., 2004). However, the joint effect of valence and arousal on recognition has been demonstrated in numerous neuroimaging studies, suggesting that these two dimensions influence memory through distinct neural mechanisms (Dolcos et al., 2004; Kensinger and Corkin, 2004). These results therefore justify the importance of considering these two dimensions in studies exploring the impact of emotions on memory.

Interestingly, prior investigations suggest that the emotional characteristics of stimuli may shape the retention of information differently over long delays, compared with short delays. In some studies, emotional enhancement was observed after a long delay, but not a short delay (Yonelinas and Ritchey, 2015). In other cases, the effects of arousal on memory were enhanced after a long delay, relative to a shorter one (Sharot and Phelps, 2004; Payne et al., 2008; Sharot and Yonelinas, 2008; Kensinger, 2009). Finally, Waring and Kensinger (2009) reported a complex interaction between all these factors in a visual scene recognition task. After a short delay, they showed that the level of arousal modulated the effect of valence on memory. Thus, an emotional enhancement of memory was observed in all conditions, except for positive low-arousal scenes, at least in young adults. After a long delay, the pattern of results was different. The enhancement of memory was greater for negative than for positive scenes, and greater for arousing than for non-arousing scenes underlying once more the negativity bias and the effect of arousal in emotional memory. Furthermore, the emotional enhancement effect was greater after a long delay than a short delay, emphasizing the role of post-encoding processes in memory consolidation of emotional information. Taken as a whole, extant literature underlines the complex interplay between emotional features of stimuli and study-test intervals, and how memory performance can be shaped by post-encoding actions.

An absence of agreement persists among musical domain scholars about how these factors contribute to the emotional memory for music. Indeed, as an enjoyable human activity that is present in all cultures, capable of generating strong and varied emotions, it seems to be a privileged medium for studying this effect. One series of studies used the exact same prototypal clips intended to express happy, fearful, peaceful, or sad emotions, emotional characteristics which had been validated previously (Gosselin et al., 2005; Vieillard et al., 2008). Musical recognition of these four emotional categories of computer-generated MIDI musical excerpts was generally assessed after a short delay (Aubé et al., 2013; Vieillard and Gilet, 2013; Narme et al., 2016), though sometimes after a longer delay of 24 h (Samson et al., 2009). Except for one study that did not report any difference in explicit recognition, at least in young adults (Narme et al., 2016), the other investigations showed better recognition of musical clips expressing fear and, to some extent, happiness (Samson et al., 2009; Aubé et al., 2013; Vieillard and Gilet, 2013), suggesting a benefit for high-arousal stimuli. However, this finding should be interpreted cautiously, as it could be explained by different levels of difficulty between recognition of high and low-arousing musical excerpts, or of each prototypical emotional category. As demonstrated by a control study (Samson et al., 2009), the perceptual distinctiveness (or dissimilarity) between the scary music was larger than the distinctiveness between the three other categories, explaining, at least in part, the superior recognition scores obtained with scary musical excerpts. However, the high recognition score obtained with happy musical excerpts, in particular after a 24-h delay, might nonetheless reflect the effect of arousal and/or valence on consolidation of musical memory, though it is too early to draw any firm conclusions.

Other studies reported in the literature have used instrumental classical music. Eschrich et al. (2005) examined the relationship between emotion and music recognition. In this study, piano pieces by Bach were rated in terms of arousal (from very pacifying to very arousing) and valence (from negative to positive valence) during encoding. Recognition tests conducted 2 weeks later revealed that well-recognized pieces were associated with higher arousal ratings and received a higher positive valence rating. In a subsequent study, symphonic film music eliciting positive feelings (i.e., little positive to very positive) were repeated twice over different days (Eschrich et al., 2008). The authors confirmed an effect of valence on recognition with better memory for very positive music excerpts than for less positive ones, although no effect of arousal on recognition was obtained in this case. These findings suggest that emotional valence, as rated by the participants, appears to influence long-term memory for music, underlying once more the impact of positive valence music on memory consolidation. However, the lack of negative valence music in this study limits the interpretation of the results. By addressing again this question in a neuroimaging study, the authors failed to replicate their previous finding (Altenmüller et al., 2014). Participants’ single exposure to the musical pieces during encoding may have been insufficient to induce a memory enhancement, thereby explaining the lack of behavioral results. Based on all these data, it remains unclear whether only positive valence or both positive and negative valence provides a memory advantage.

Prior investigations into emotional memory in music have assessed memory at only one delay. To our knowledge, in the music domain, only one study has examined the effects of delay interval upon emotional memory (Alonso et al., 2015). Making use of the parsimonious model of emotion, which defines emotional spectrum into two dimensions, its authors manipulated valence and arousal levels in symphonic musical excerpts. Participants were requested to rate these two emotional feelings induced by listening to music before taking two recognition memory tests administered immediately after the study and 24 h after the study session. The results indicated that arousal and valence interacted differently with memory performance at each study-test delay. In immediate recognition, the effect of valence varied as a function of arousal. Whereas valence did not interfere with the remembering of high-arousal excerpts, it did modulate recognition of low-arousal stimuli such that positive excerpts were better recognized than negative excerpts. In contrast, in the delayed condition, the results revealed no interaction between the two emotional dimensions. Only independent effects of arousal and valence were reported, such that high-arousal excerpts and negative excerpts were better memorized than low-arousal and positive ones, respectively, confirming the memory advantage for high arousing and negative stimuli already reported in non-musical domains (Ochsner, 2000; Waring and Kensinger, 2009). Unlike findings obtained with words and pictures, there was no loss of memory in delayed, as compared to immediate recognition, indicating no deleterious impact of delay interval in music. Yet this recognition test, frequently used in psychology, presents a methodological bias. By presenting the target stimuli once again in the delayed recognition test, this condition benefits from an additional exposure compared to the immediate condition. It is therefore difficult to disentangle the effect of delay from the effect had by number of presentations in post-encoding processes.

To overcome this methodological limit and to clarify the ways emotional dimensions (i.e., valence and arousal) affect musical memory as study-test delay intervals increase, we designed a new study using the symphonic musical excerpts selected by Alonso et al. (2015). However, we manipulated the study-test interval while keeping the number of exposures constant. For this purpose, the encoding phase was distributed over two distinct sessions, one session on day 1 allowing the first half of the target stimuli to be encoded and the other session on day 2 allowing the other half to be encoded. Immediately after this second session on day 2, a recognition test including all target stimuli mixed with foils was presented. The recognition of the target stimuli presented just before (on day 2) or after 24 h (on day 1) provided respective memory performances after a short delay and a long-delay retention without changing the number of exposures to the targets. In line with the literature, we hypothesized an emotional enhancement of music memory induced by post-encoding processes leading to better recognition of musical excerpts in delayed condition as compared to immediate one. The effects of arousal and valence should become exaggerated after a long delay. Finally, we also predicted that the two emotional dimensions would be differently affected by the study-test intervals.

## Materials and Methods

### Participants

Eighty native French speakers took part in this study (mean age = 34.09 ± 7.20; 37 females and 43 men). In accordance with the Music Expertise Questionnaire (Ehrlé, 1998), 43 participants had no musical expertise and 37 were musicians. The quality and quantity of sleep was also assessed twice using the St. Mary’s Hospital Sleep Questionnaire (Ellis et al., 1981; mean duration of sleep, night 1 = 7.48 ± 1.61, night 2 = 7.57 ± 1.41). No participants reported a history of psychiatric or neurological disorders, alcoholism, or present treatment with centrally acting medications. Participants were screened for the presence of mood disorders using the Profile of Mood State (POMS; Shacham, 1983; mean score for anxiety = 4.42 ± 3.57, depression = 2.65 ± 2.55, confusion = 2.00 ± 3.79, anger = 4.03 ± 3.15, tiredness = 6.15 ± 3.70, and vigor = 11.60 ± 3.55) and for the presence of attention deficit using standardized psychometric tests: the Paced Auditory Serial Addition Test (PASAT; Mazza and Naegele, 2004; mean score = 49.66 ± 6.15) and the Wechsler adult intelligence scale–Fourth Edition (WAIS-IV) coding subtest (Wechsler, 2014; mean scaled score = 11.25 ± 12.67). All participants have signed informed consent and the study was carried out following the Declaration of Helsinki principles.

### Stimuli

The musical material consisted of 32 symphonic excerpts with a duration of 5 s (±1-s fade and in fade out) already used in a previous study (Alonso et al., 2015). These excerpts were taken from different symphonies written by composers between 1830 and 1954 and were normalized to a maximal amplitude of 1.2 dB. To control the effect of familiarity, the most famous composers and symphonies of this period were excluded. Musical stimuli had been previously rated for stimulus valence (positive vs. negative) and arousal levels (high vs. low) to create four different emotional combinations in a two by two design: high-arousal and positive (A+; V+); high-arousal and negative (A+; V−); low-arousal and positive (A−; V+); and low-arousal and negative (A−; V+). We selected 16 target stimuli (four per emotional combination) and 16 distractors for the recognition test. Thus, to each target was matched to a distractor from the same emotional combination and melodic style, composed by the same composer or even excerpts from the same symphony as the target (the list of the symphonies is presented in Alonso et al., 2015).

### Procedure

Participants sat in front of a laptop in a quiet room and listened to the musical excerpts using stereophonic headphones. PsychoPy software was used to run the experiment and to record recognition ratings. All participants were tested individually over two different sessions, with the encoding phase being split over two consecutive days. On day 1, they listened to eight different musical excerpts (two stimuli per emotional combination). Participants were instructed to listen carefully to the musical excepts because their memory would be tested later (intentional encoding). To reinforce the encoding process, the studied items were presented three times in random order. Two questionnaires were administered: the St. Mary’s Hospital Sleep Questionnaire (Ellis et al., 1981) and the POMS (Shacham, 1983).

On day 2, approximately 24 h later, each participant listened to eight additional musical excerpts following the same procedure used on day 1. As with the first encoding phase, the items were presented three times. On both days, participants completed questionnaires either before or after the encoding task in a randomized manner, on day 2 the St. Mary’s Hospital Sleep Questionnaire (Ellis et al., 1981) and the Music Expertise Questionnaire (Ehrlé, 1998) were administered. Then, 15 min after the second encoding phase, the recognition phase began. Sixteen targets, including eight musical excerpts encoded on day 1 and eight musical excerpts encoded on day 2, were randomly intermixed with 16 distractors. Participants were told that items from the same-day study session and the study session a day earlier were intermixed with novel items. This recognition phase assessed memory recognition after short (15-min) and long (24-h) delays within a single test. After each presentation, participants had to decide whether they had already heard a given excerpt (yes/no) and to assign their responses confidence ratings (sure/unsure). Participants were not given a time limit to respond. During the 15-min delay, the participants performed standardized cognitive tests to assess auditory attention and working memory using the PASAT (Mazza and Naegele, 2004) and the WAIS–IV coding subtest (Wechsler, 2014).

### Data Analysis

Based on the accuracy of the responses and the confidence ratings, receiver operating characteristic (ROC; Yonelinas, 1994) curves have been calculated for each arousal and valence combination. Preliminary analyses were carried out to test the effects of age, sex, musical expertise and duration of sleep on the global recognition score. Then, a three-way repeated-measure analysis of variance (ANOVA) with arousal (high/low), valence (positive/negative), study-to-test delay period (short/long) as well as subsequent *post hoc* analysis was carried out.

## Results

### Preliminary Analysis

These analyses showed there was no correlation between the recognition of the musical material and participants’ age (*r* = −0.201; *p* = 0.073) or duration of sleep (*r* = −0.075; *p* = 0.510). Furthermore, we found sex and musical expertise not to have an effect on the recognition test (all *ps* > 0.05). Finally, the completion of the questionnaires before or after the encoding tasks had no effect on the musical recognition (*U* = 744; *df* = 78; *p* = 0.593).

### Recognition Task

**Figure 1:**
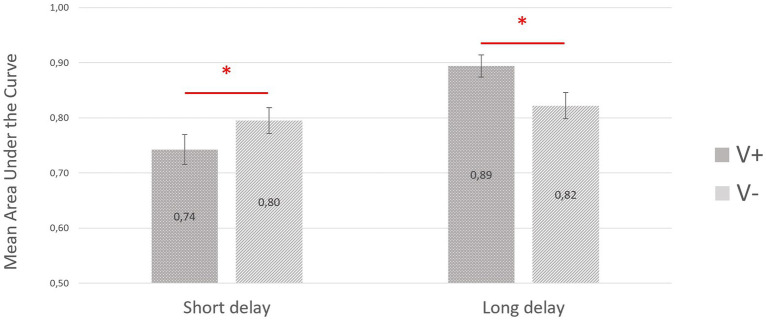
Mean areas under the receiver operating characteristic (ROC) curve for the recognition of positive and negative musical excerpts as a function of delay (short vs. long). Origin is set at chance level (0.50). The error bars correspond to the standard error of the mean.

The ANOVA with three within-subjects factors (i.e., valence, arousal, and delay) was carried out on the areas under the ROC curves. The analysis showed a valence by delay interaction (*F*_(1,79)_ = 23.74; *p* < 0.001; *η_p_*^2^ = 0.231), as depicted in Figure 1. *Post hoc* analyses showed that after a short delay, the mean area under the curve was higher for the negatively valenced (mean ± SD = 0.80 ± 0.24) than for positively valenced excerpts (mean ± SD = 0.74 ± 0.24; *p* < 0.05). In contrast, after a long delay, the mean area under the curve was higher for positively valenced (mean ± SD = 0.89 ± 0.18) than for negatively valenced excerpts (mean ± SD = 0.82 ± 0.21; *p* < 0.002).

This ANOVA also revealed a main effect of delay (*F*_(1,79)_ = 43.63; *p* < 0.001; *η_p_*^2^ = 0.356), with the mean area under the curve being significantly higher after long delay (mean ± SD = 0.85 ± 0.11) than after short delay (mean ± SD = 0.76 ± 0.13). No main other effect was had by arousal (*F*_(1,79)_ = 0.08; *p* > 0.05), valence (*F*_(1,79)_ = 0.39; *p* > 0.05), or interaction.

## Discussion

The objective of this study was to investigate the impact of emotional dimensions on musical memory by comparing the effects of valence (i.e., positive and negative) and arousal (i.e., low and high) on the recognition of musical excerpts. To improve our understanding about how emotional dimensions (i.e., valence and arousal) affect musical memory as study-test delay intervals increase, we compared memory performance after a short (15 min) and a long (24 h) retention delay. We showed that the emotional enhancement of memory depends upon valence, with musical memory of positive and negative stimuli being differently affected by the duration of the study-test delay interval. Furthermore, our data demonstrated that musical excerpts were better recognized after a long delay than after a short delay, illustrating that memory consolidation for musical information is taking place during the long study-test interval.

The main finding borne out by our study is that emotion strengthens musical memory. More specifically, we showed that emotional valence differently affected the recognition of musical excerpts after a short‐ and a long-delay interval. Whereas remembering of musical excerpts is higher for negative than for positive stimuli after a short delay, the reverse was observed after a long delay. Thus, the memory performance for stimuli associated with positive valence was not only higher than for negative ones, but also improved after a 24-h delay, which was not the case for the negative stimuli. The fact that emotional enhancement on musical memory varies as a function of study-test delay intervals confirmed our predictions and is compatible with previously reported results (Alonso et al., 2015). It suggests that post-encoding processes, including consolidation that transforms newly formed memories from a fragile state to a more permanent state, modulated the effects of emotion upon musical memory. However, the discrepancies between the pattern of results obtained in the current study and that of our previous study with Alonso et al. (2015) raised several questions worth discussing.

By examining the impact of aging on emotional musical memory, Alonso et al. (2015) found that valence interacted with arousal when recognition was assessed after a short delay. Whereas valence had no impact on the recognition of high-arousal stimuli, it did affect low-arousal stimuli, with positive valence music being better remembered than negative valence music. After a long delay, only the effects of valence and arousal were demonstrated, with negative valence stimuli being better recognized than positive ones, and high-arousal stimuli better recognized than low-arousal stimuli. The different result profiles obtained after short and long-delay intervals in Alonso et al.’s study and in the present study are surprising, since both used the exact same set of stimuli. The apparent discrepancies between the studies might nonetheless be due to methodological differences. In Alonso et al. (2015), ratings of emotional dimensions were requested during the first exposure to the musical excerpts, whereas in the present study, no specific instructions were given at the time of encoding. By asking participants to rate valence and arousal, attention devoted to the emotional meaning of the stimuli might have induced deeper encoding processes than in the present study. As previously discussed in the literature (Everaert et al., 2011; Greenberg et al., 2012), when participants are particularly attentive to emotion, the effects of arousal on memory are more likely to appear. Such a memory enhancement for arousing stimuli can be even greater after long study-test delay periods than after short ones (Sharot and Phelps, 2004; Sharot and Yonelinas, 2008), at least when emotional stimuli (i.e., words or pictures) were compared with neutral stimuli. Taken as a whole, these results underline the importance of the depth of encoding in emotional memory. Unlike verbal or visual information, the concept of neutral emotion does not really exist in music, which is intrinsically emotional. Thus, music is defined as the language of emotion and is rarely perceived as non-emotional. Rather than “neutral” music, Dellacherie et al. (2008) have proposed “ambiguous” music, that is, when the emotional cues are insufficient to evoke a specific emotion. Since the concept of neutral emotion in music is questionable, we deliberately used only emotional musical excerpts in the current study as in Alonso et al. (2015). However, a fruitful avenue for future research may be to modify the encoding instructions in order to improve use of controlled cognitive mechanisms and provide an opportunity for sustained attention effects to influence memory. It is possible that under these conditions, an effect from arousal on musical memory would be noted, as in Alonso et al. (2015) and Eschrich et al. (2005), who both asked participants to judge arousal and valence at encoding during an intentional memory task.

Another important difference between the present investigation and Alonso et al. (2015) concerns procedure. In Alonso et al. (2015), all target stimuli were presented within a single session on day 1, followed by two recognition tests, one proposed after a short delay and the other after a long delay. In this case, as in many classical recognition paradigms, delayed recognition performance benefited not only from a longer consolidation time but also from an additional exposure to the target stimuli, relative to immediate recognition. To overcome this methodological bias in the present study, we split the presentation of the target stimuli into two study phase sessions, with half of them being presented on day 1 and the other half on day 2. The memory test presented on day 2 after the second study phase allowed us to test recognition after a short and a long delay while keeping the number of exposures constant. Instead of making participants learn the whole series of 16 target stimuli and apply them in a single block presentation, we distributed the 16 targets across two sessions, each with only eight targets. Finally, Alonso et al. (2015) investigated a group of young adults (mean age = 22 years) with a group of elderly people (mean age = 75 years). Since they found no effect of age on performance, they mixed all the 30 participants. As a result, their participants displayed a larger age range and were globally older than participants from the present study. Despite these methodological differences in terms of the number of items to be learned, the number of exposures, the different instructions and subjects’ characteristics, it appears that negative valence stimuli were similarly recognized after long delay in both studies (ROC = 0.82 in the present study; ROC = 0.80 in Alonso et al., 2015). However, positive valence stimuli were better recognized in the present study (ROC = 0.89) than in Alonso et al. (2015) (ROC = 0.74). This finding firstly indicates that the consolidation process improves musical memory when the number of stimuli to be learned is limited. Secondly, the decrease of performance in recognizing positive valence stimuli in Alonso et al. (2015) can also be explained by the age of the participants. However, this hypothesis seems rather improbable since the age does not seem to affect the recognition of negative valence stimuli. Moreover, it would contradict the positivity bias found in the aging process, which is described in the literature (for review, see Reed et al., 2014). Finally, the advantage of positive valence over negative valence on recognition performance after a long-delay interval in the present study underlines the importance of emotional valence in memory consolidation of music. This final result seems to be in agreement with Eschrich et al. (2008) who showed a better recognition, after 48 h, of very positive music compared to less positive music. Thus, our study, by responding to the limitations of Alonso et al. (i.e., the difference in number of presentations, the heterogeneous population, and the small sample size) brings new evidence on the impact of the emotional dimensions and in particular the positive valence on the consolidation of musical memory.

The better consolidation of positive vs. negative stimuli reported here suggests that emotion does not have a uniform effect on memory. Moreover, it is noteworthy that previously reported studies also demonstrated a positive valence advantage on musical memory after long time periods (after a 24-h delay for Samson et al., 2009 and after several days or weeks for Eschrich et al., 2005, 2008). In these cases as well, memory was assessed at only one delay interval. Furthermore, this result confirms that memory enhancement is particularly pronounced over time (Yonelinas and Ritchey, 2015). While the memory traces of neutral information are gradually affected by a phenomenon of forgetfulness (Ebbinghaus, 1885), emotional stimuli are more resistant and even better recognized after a long period of time thanks to the consolidation process. Emotions generated by the musical material make musical memory more resistant to forgetting, a finding consistent with the literature on the emotional enhancement effect of memory (Jäncke, 2008). The novel aspect of this current study, however, is the finding that musical memory consolidation is strongly related to the characteristics of the positive valence, which is not the case in studies in the non-musical field. Using pictures and words, several studies demonstrated a negative valence effect on memory (for review, see Kensinger, 2007). Indeed, according to evolutionary theories, the main function of emotion would be to guide our behavior: fear, for example, allows us to avoid danger and prepares us to act (Darwin, 1872). It is therefore logical that our attention should focus more on the threatening elements, thus allowing us to better memorize negative information (Kensinger, 2007). Given that music is a predominantly hedonic activity, this negativity bias would not be found for musical stimuli.

Another particularity of the current study is to have used exclusively non-familiar musical excerpts to avoid any confounding factors related to familiarity. Indeed, when musical excerpts are known, such as the piano pieces by Bach used in the study by Eschrich et al. (2005), the emotion associated with the excerpts would be more often linked to an autobiographical event (Janata et al., 2007). As a result, these excerpts could lead participants to remember memories and convey more intense emotions than when faced with unfamiliar music. This could also explain the effect of the arousal found in the study by Eschrich et al. (2005) that we were not able to demonstrate in the current study. Although we were not in control of the encoding strategies used by the listeners, we can assume that performing an elaborative rehearsal with non-familiar music is more complex, and probably impossible. Unlike pictures or words, which are generally associated with preexisting knowledge, new pieces of music are difficult to relate to already existing information. In this case, difficulties in thinking about the meaning of an item to be remembered, or making connections between that item and prior knowledge, may have limited the use of elaborative rehearsal known to improve memory abilities. Therefore, when exposed to new music, listeners can only rely on their own emotional feeling, on the attractiveness or the pleasantness of the musical pieces or on their familiarity to the musical style rather than to a specific event. One recent study demonstrated musical memory to be strongly related to pleasantness (Ferreri and Rodriguez-Fornells, 2017). The authors showed that musical excerpts rated as very pleasant were better recognized than musical excerpts rated as less pleasant. Listeners may indeed experience greater pleasure from listening to positive valence music than negative valence music. Based on all previously reported findings, we therefore propose that the time-dependent effect of emotion attributed to a process of emotional consolidation is mainly predicated on the positive or pleasurable experience of music. It remains to be clarified whether dispensing instructions to rate pleasantness will further improve memory consolidation for music. Moreover, a relationship between arousal and pleasure has already been discussed by Berlyne’s hedonic model (Berlyne, 1971). According to this model, very low or high arousal values would lead to low pleasure whereas medium arousal values would lead to high pleasure (Berlyne, 1971). This suggests the existence of a complex relationship between arousal and pleasure that needs to be further investigated in the musical domain. Another point to be clarified in the future would be the impact of musical characteristics that might have contributed to emotion and to musical memory. For instance, it has been reported that variations in intrinsic musical characteristics such as timbre or tempo modulate musical memory (Halpern and Müllensiefen, 2008). Even if the musical excerpts used in our study were very homogeneous in terms of musical characteristics, such as rhythm, mode, and tempo since targets and distractors were selected from small set symphonies written by composers between 1830 and 1954, we cannot exclude the impact of intrinsic musical features in recognition.

To conclude, the present study succeeded in demonstrating the impact of emotion on musical long-term memory, thus improving our understanding of the specific role played by emotional valence and arousal on memory consolidation. The positivity effect observed in young adults after a long study-test interval may be related to the effect of pleasantness in remembering musical excerpts recently reported in the literature (Ferreri and Rodriguez-Fornells, 2017), lending credence to arguments in favor of considering memory differently according to valence (i.e., positive vs. negative). To our knowledge, our study is the first to demonstrate a clear improvement in remembering positive musical excerpts resulting from a consolidation process. Further study will be necessary to generalize this emotional enhancement of musical memory to other musical styles and different populations, from children to the elderly. If positive music has such a strong influence on our cognitive system and in particular on our memory abilities, it raises questions as to whether the memory-enhancing effect of emotional music could be used to enhance cognitive performance in general and clinical settings.

## Data Availability Statement

The raw data supporting the conclusions of this article will be made available by the authors, without undue reservation.

## Ethics Statement

Ethical review and approval was not required for the study on human participants in accordance with the local legislation and institutional requirements. The patients/participants provided their written informed consent to participate in this study.

## Author Contributions

CN, DD, and SS contributed to the design and implementation of the research, to the analysis of the results, and to the writing of the manuscript. All authors contributed to the article and approved the submitted version.

### Conflict of Interest

The authors declare that the research was conducted in the absence of any commercial or financial relationships that could be construed as a potential conflict of interest.
